# An Allele Real-Coded Quantum Evolutionary Algorithm Based on Hybrid Updating Strategy

**DOI:** 10.1155/2016/9891382

**Published:** 2016-01-10

**Authors:** Yu-Xian Zhang, Xiao-Yi Qian, Hui-Deng Peng, Jian-Hui Wang

**Affiliations:** ^1^School of Electrical Engineering, Shenyang University of Technology, Shenyang 110870, China; ^2^School of Information Science and Engineering, Shenyang University of Technology, Shenyang 110870, China; ^3^College of Information Science and Engineering, Northeastern University, Shenyang 110004, China

## Abstract

For improving convergence rate and preventing prematurity in quantum evolutionary algorithm, an allele real-coded quantum evolutionary algorithm based on hybrid updating strategy is presented. The real variables are coded with probability superposition of allele. A hybrid updating strategy balancing the global search and local search is presented in which the superior allele is defined. On the basis of superior allele and inferior allele, a guided evolutionary process as well as updating allele with variable scale contraction is adopted. And *H*_*ε*_ gate is introduced to prevent prematurity. Furthermore, the global convergence of proposed algorithm is proved by* Markov* chain. Finally, the proposed algorithm is compared with genetic algorithm, quantum evolutionary algorithm, and double chains quantum genetic algorithm in solving continuous optimization problem, and the experimental results verify the advantages on convergence rate and search accuracy.

## 1. Introduction

Quantum computing is a new class of computing algorithms based on the concepts and principles of quantum theory, such as superposition of quantum states, entanglement, and intervention. Quantum evolutionary algorithm (QEA) is proposed by combining quantum theory with evolutionary theory. It is a kind of evolutionary algorithm with the form of quantum chromosome; the random observation which simulates the quantum collapse can bring diverse individuals [1]. In QEA, the quantum bits (Q-bits) and quantum gate (Q-gate) have been used to update the individuals to realize evolutionary search. QEA has advantages, including the diversity of population, global search performance, and convergence rate, compared with other evolutionary algorithms due to Q-bits coding and the updating of rotation gate. Since then, QEA were developed, including different operators [2–5] and novel update strategy for Q-gates [6–9]. QEA has been applied in many optimization problems [10, 11]. However, frequent encoding and decoding in QEA will slow down convergence rate. In addition, the magnitude and the direction of the rotational angle of quantum rotation gates cannot be determined during optimization.

Recently, numerous literatures on QEA have been reported. Han and Kim [12] proposed a quantum-inspired evolutionary algorithm with *H*_*ε*_ gate in which the probability amplitudes of rotation gate converge to *ε* and 1 − *ε*. Chen et al. [13] proposed a rotation gate with chaos in QEA. It applied generated chaotic sequence beforehand at the updating of rotation gate to improve the computational speed. Yang et al. [14] proposed a rotation gate based on arithmetic guidance. In the proposed algorithm, the current optimal individuals transfer to next generation. S. Y. Li and P. C. Li [15] proposed the double chains quantum genetic algorithm (DCQGA) which mapped the real variables to the probability amplitude on Q-bit by transforming the solution space; in particular, a real Q-bit encoding can be used to avoid binary coding. da Cruz et al. [16] proposed a real-coded quantum evolutionary algorithm in which the solution space is expressed by rectangular region. Its outstanding performance in solving high dimensional continuous optimization problems was verified. P. Li and S. Li [17] proposed a Bloch spherical coding for QEA which can avoid local optimization in solving continuous optimization problems. Gao et al. [18] proposed a real-coded quantum evolutionary algorithm based on triploid coding. Zhang et al. [19] proposed four-chain quantum-inspired evolutionary algorithm in which a chromosome contains four gene chains. The above quantum evolutionary algorithms introduce Q-bit encoding and increase diversity of population but spend much time on encoding and decoding; meanwhile, the search process may fall into local minimum and prematurity. So it is very important for us to employ a novel updating strategy in order to improve convergence rate and prevent the premature.

In this paper, we presented an allele real-coded quantum evolutionary algorithm (ARQEA) based on hybrid updating strategy. The proposed algorithm employs a real-coding to maintain the diversity of allele and introduces a hybrid updating strategy to balance performance between global search and local search in order to improve the convergence rate. The convergence of ARQEA is verified by* Markov* chain. The numerical examples verify the advantages of ARQEA on convergence rate and search accuracy.

The remainder of this paper is organized as follows: in Section 2, an allele real-coded quantum evolutionary algorithm is proposed in which the real coding as well as hybrid update strategy is presented; in Section 3, the global convergence of proposed algorithm is verified by* Markov* chain. Finally, the numerical experiments are demonstrated and the advantages are verified in Section 4.

## 2. An Allele Real-Coded Quantum Evolutionary Algorithm

In ARQEA, the real variables are coding with probability superposition of allele. A hybrid evolutionary strategy balancing the global search and local search is presented in which the superior allele is defined. The supervised evolution as well as variable scale contraction is adopted, and *H*_*ε*_ gate is introduced to prevent the premature convergence.

### 2.1. Real Coding for Allele

We present an allele real coding which will generate two continuous variables *x*_*i*_ and *x*_*i*_′ (*x*_*i*_ and *x*_*i*_′ are defined as the *i*th alleles) to replace 0 and 1 gene of Q-bit coding in QEA. The real coding is

(1) $$\begin{array}{c} \left|\begin{array}{cccccc} x_{1}^{} & x_{2}^{} & \cdots & x_{i}^{} & \cdots & x_{n}^{}\\ x_{1}^{\prime } & x_{2}^{\prime } & \cdots & x_{i}^{\prime } & \cdots & x_{n}^{\prime } \end{array}\right|, \end{array}$$

where *n* is the number of variables.

The probability amplitudes of allele are expressed as trigonometric functions cos *θ*_*i*_ and sin *θ*_*i*_:

(2) $$\begin{array}{c} \left|\begin{array}{cccccc} \cos\theta_{1} & \cos\theta_{2}^{} & \cdots & \cos\theta_{i}^{} & \cdots & \cos\theta_{n}^{}\\ \sin\theta_{1} & \sin\theta_{2} & \cdots & \sin\theta_{i}^{} & \cdots & \sin\theta_{n}^{} \end{array}\right|, \end{array}$$

where cos *θ*_*i*_ and sin *θ*_*i*_ are the probability amplitudes of *x*_*i*_ and *x*_*i*_′; the initial value of *θ*_*i*_ is *π*/4, *i* = , 1,…, *n*.

Then the observed probability of *x*_*i*_ and *x*_*i*_′ is cos^2^⁡ *θ*_*i*_ and sin^2^⁡ *θ*_*i*_, respectively. So

(3) $$\begin{array}{c} \cos^{2}\theta_{i}+\sin^{2}\theta_{i}=1. \end{array}$$

We employ the probability superposition to maintain the diversity for individual in real coding. And the proposed real coding prevents overlong chromosome length and saves time spent on encoding and decoding.

### 2.2. Hybrid Updating Strategy

In ARQEA, we adopt hybrid updating strategy to update allele and its probability amplitudes.

#### 2.2.1. Updating Alleles

**Figure 1:**
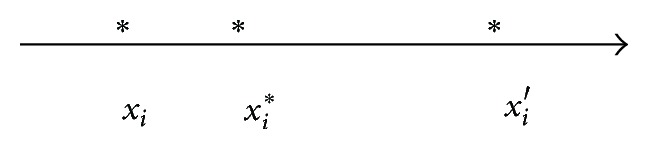
The superior and inferior alleles.

Firstly, we encode initial population on the basis of allele real coding scheme, compute the fitness of chromosomes, and recode the superior individual *x*_*i*_^*∗*^. Then we compare the distances between the *i*th alleles *x*_*i*_ and *x*_*i*_′ and define the allele corresponding to the shorter distance with *x*_*i*_^*∗*^ as “superior allele,” the other one as “inferior allele.” Figure 1 shows the superior and inferior alleles, in which *x*_*i*_ and *x*_*i*_′ are the *i*th alleles of chromosome, *x*_*i*_^*∗*^ is the *i*th allele of the current optimal individual, and then we define *x*_*i*_ as the superior allele and *x*_*i*_′ as the inferior allele.

Next, we will update the alleles. The updating strategy is described as follows:

(a) For the superior allele, we employ the updating strategy, such as reasonable choice of the initial step length and dynamic adjustment of the search step length, to guide superior allele to search optimal solution.

In the early search stage, the updating strategy is accelerated by directing the search direction

(4) $$\begin{array}{c} x_{i+1}=x_{i}+\mathrm{sign}\left(\;x_{i}^{*}-x_{i}\;\right)\cdot \Delta d_{1}, \end{array}$$

where *x*_*i*_ is a superior allele, *x*_*i*+1_ is the updated allele, Δ*d*_1_ = *λ* · |*x*_*i*_^*∗*^ − *x*_*i*_| is the search step length, usually *λ* = 0.5, and sign() is a sign function; it decides the search direction of superior allele.

When |*x*_*i*_^*∗*^ − *x*_*i*_|/(*x*_max_ − *x*_min_) is less than *η*, we adopt a variable scale contraction to search the optimal solution

(5) $$\begin{array}{c} x_{i+1}=x_{i}+U\left(\;-1,1\;\right)\cdot {\left(\;1-arctan\left(\;\frac{r}{g}\;\right)\;\right)}^{4}\cdot \Delta d_{2}, \end{array}$$

where *r* is the number of current generation, *g* is the number of max generation, *U*(−1,1) is a random distribution between −1 and 1, (1 − arctan(*r*/*g*))^4^ is a change from 1 to 0 with the number of generation *r*, and Δ*d* is the range of contraction. When *U*(−1,1) is a negative number, Δ*d*_2_ = *x*_*i*_ − *x*_min_.

In this search stage, the superior allele will randomly search the optimal solution in a certain region, and the searching scale will change with the number of generation *r*.

(b) For the inferior allele *x*_*i*_′, the allele *x*_*i*_ will be substituted to *x*_*i*_′ in (4) and (5) and alternate operation every two generations using (4) and (5).

However, considering the randomness of the evolutionary algorithm, the inferior allele has the ability to escape from local optimum and approach the global optimum in the evolution stage.

#### 2.2.2. Updating Probability Amplitudes

After updating the allele, *H*_*ε*_ gate is used to update the corresponding probability amplitudes [20, 21]. We employ *H*_*ε*_ gate operation to prevent the premature convergence. The rotation angle of *H*_*ε*_ gate is

(6) $$\begin{array}{c} \theta_{i+1}=\theta_{i}\pm {\left(\;1-\arctan\left(\;\frac{r}{g}\;\right)\;\right)}^{4}\Delta t, \end{array}$$

where Δ*t* is the range of *θ*_*i*_ and its direction depends on the superior allele.

If the superior allele corresponds to cos *θ*_*i*_, then (6) is subtracting. Instead, it is adding. The *H*_*ε*_ gate operation will increase the observed probability for the superior allele and decrease the observed probability for the inferior allele.

The operation rule of *H*_*ε*_ gate is the following:

(a) if |*α*_*i*_′|^2^ ≤ *ε* and |*β*_*i*_′|^2^ ≥ 1 − *ε*, then ${\left[\begin{array}{cc} \alpha_{i}^{\prime \prime } & \beta_{i}^{\prime \prime } \end{array}\right]}^{T}={\left[\begin{array}{cc} \sqrt{\varepsilon } & \sqrt{1-\varepsilon } \end{array}\right]}^{T}$;
(b) if |*α*_*i*_′|^2^ ≥ 1 − *ε* and |*β*_*i*_′|^2^ ≤ *ε*, then ${\left[\begin{array}{cc} \alpha_{i}^{\prime \prime } & \beta_{i}^{\prime \prime } \end{array}\right]}^{T}={\left[\begin{array}{cc} \sqrt{1-\varepsilon } & \sqrt{\varepsilon } \end{array}\right]}^{T}$;
(c) else ${\left[\begin{array}{cc} \alpha_{i}^{\prime \prime } & \beta_{i}^{\prime \prime } \end{array}\right]}^{T}={\left[\begin{array}{cc} \alpha_{i}^{\prime } & \beta_{i}^{\prime } \end{array}\right]}^{T}$,

where *ε* is a real number between 0 and 1, $\left[\begin{array}{cc} \alpha_{i}^{\prime } & \beta_{i}^{\prime } \end{array}\right]$ are the probability amplitudes after the *H*_*ε*_ gate operation, and $\left[\begin{array}{cc} \alpha_{i}^{\prime \prime } & \beta_{i}^{\prime \prime } \end{array}\right]$ are the probability amplitudes after the *H*_*ε*_ gate operation.

The steps of hybrid evolutionary strategy are described as follows.

**Step 1 .**

Judge the relative superior allele.

**Step 2 .**

For the superior allele, adopt supervised evolution by (4) to find current optimal allele *x*_*i*_^*∗*^, and employ variable scale contraction by (5) approach to local search when |*x*_*i*_^*∗*^ − *x*_*i*_|/(*x*_max_ − *x*_min_) is less than *η*.

**Step 3 .**

For the inferior allele, search alternately by (4) and (5) in every two generations.

**Step 4 .**

Update the probability amplitudes of alleles by (6).

In hybrid updating strategy, we employ different evolutionary mode for superior and inferior allele in order to balance global search and local search. The superior allele affects local search stage, and inferior allele plays important role in global search by the variable scale contraction to search unknown space and maintain the diversity of population. As generation goes on, superior allele and inferior allele exchange position in next generation dynamically. The hybrid updating strategy proposed has two mechanisms for superior and inferior allele to maintain diversity of population. In addition, the hybrid updating strategy in ARQEA balances the global search and local search by dynamic updating strategy of alleles.

### 2.3. Process of ARQEA

**Figure 2:**
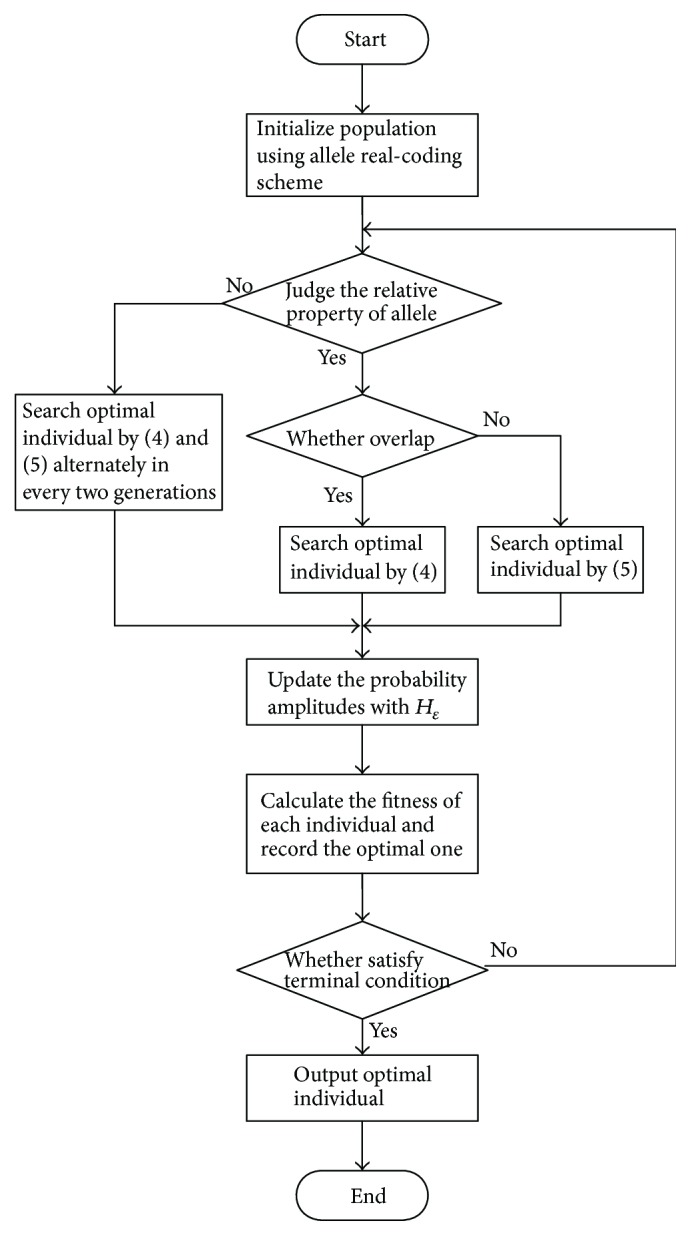
The flowchart of ARQEA.

ARQEA employs allele real coding with probability superposition which generates a random number *r*_*i*_ between 0 and 1 and compares *r*_*i*_ with the probability amplitudes for each allele and adopts hybrid updating strategy to balance global search and local search. At the early search stage, the scale of mutation is large, the step size in search is long, and the algorithm approaches to the global search. With the increasing of generations, the scale of mutation becomes smaller and the performance of local search is strengthened. The process of ARQEA is shown in Figure 2.

## 3. Convergence Analyses

In this section, we analyze the global convergence of ARQEA by* Markov* chain. The ARQEA is described as follows.

Consider ARQEA:

(7) Ω,G,D,P,n,X0,X′t,X′′t,Xt,Fmin,Xbest,Fbest,ε,

where *Ω* is search space, *G* is the evolutionary operator of superior allele, *D* is the evolutionary operator of inferior allele, *P* is the operator of probability selection, *n* is the dimension of *Ω*, *X*(0) is initial population, *X*′(*t*), *X*′′(*t*), and *X*(*t*) are the superior allele, the inferior allele, and the observed allele in *t*th generation, respectively, *F*_min_ is the fitness function, *X*_best_ is the optimal solution and *F*_best_ is optimal value of the fitness function, and *ε* is the termination criterion.

According to the hybrid update strategy, the population sequence of ARQEA can be expressed as follows:

(8) $$\begin{array}{c} X\left(\;t+1\;\right)=PG\left(\;X^{\prime }\left(\;t\;\right)\;\right)+\left(\;1-P\;\right)D\left(\;X^{\prime \prime }\left(\;t\;\right)\;\right). \end{array}$$

In (8), *X*(*t* + 1) relates to the state of generations *t*.

Then, the state space of ARQEA can be expressed as

(9) $$\begin{array}{c} \Omega =\left\{\;\Omega_{1},\Omega_{2},\ldots ,\Omega_{max}\;\right\}, \end{array}$$

where max = ∏_*i*=1_^*n*^((*X*_*i*_^*u*^ − *X*_*i*_^*d*^)/*ε*); *X*_*i*_^*u*^ and *X*_*i*_^*d*^ are the upper bound and lower bound of the *i*th variable. So ARQEA can be regarded as a finite* Markov* process.

Then the state space *Ω* is divided into two search spaces according to the termination criteria:

(a) *S*_1_ = {*Ω*_*i*_∣|*F*_*s*_*i*__ − *F*_best_| < *ε*};
(b) *S*_2_ = {*Ω*_*i*_∣|*F*_*s*_*i*__ − *F*_best_| ≥ *ε*}.

We define *p*_1→1_ as the transition probability from *S*_1_ to *S*_1_, *p*_2→1_ as the transition probability from *S*_2_ to *S*_1_, and *p*_2→2_ as the transition probability from *S*_2_ to *S*_2_. And *p*_1→1_ = 1.

**Lemma 3 A (see [22]).**

Assume *A*_1_, *A*_2_,… are the event sequence in probability space, and let *p* ^*k*^ = *p*{*A*_*k*_}; if ∑_*k*=1_^*∞*^ *p* ^*k*^ < *∞*, then *p*{⋂_*i*=1_^*∞*^⋃_*k*≥*i*_ *A*_*k*_} = 0; if ∑_*k*=1_^*∞*^ *p* ^*k*^ = *∞* and all *A*_*k*_ are mutually independent, then *p*{⋂_*i*=1_^*∞*^⋃_*k*≥*i*_ *A*_*k*_} = 1.

**Theorem 3 A.**

∃*φ* ∈ (0,1), make *p*_2→2_ < *φ* in ARQEA.

**Proof**

∃*d* > 0, when *ε* > 0 and |*X*(*t*) − *X*_best_| ≤ *d*, then |*F*(*X*(*t*)) − *F*_best_| < *λ*, in which 0 < *λ* < *ε*; if *C*_1_ = {*X*(*t*)∣|*X*(*t*) − *X*_best_| ≤ *d*}, then *C*_1_ ⊂ *S*_1_.

The variable scale contraction in ARQEA is Gaussian distribution. If the individual *X* in *S*_2_ performs variable scale contraction operation and obtained *X* + *δ*, then

(10) $$\begin{array}{c} p\left\{\;\left(\;X+\delta \;\right)\in C_{1}\;\right\}<p\left\{\;\left(\;X+\delta \;\right)\in S_{1}\;\right\}=p_{2\to 1}. \end{array}$$

Since *δ* is Gaussian distribution *N*(0, *δ* ^2^), the probability density is

(11) $$\begin{array}{c} f\left(\;x\;\right)=\frac{1}{\sigma \sqrt{2\pi }}\cdot e^{-x^{2}/2\sigma^{2}}. \end{array}$$

Because

(12) pX+δ∈C1∏i=1npXi+δ−Xbest≤d=∏i=1n∫Xbest−Xi−dXbest−Xi+dfxdx,

then

(13) 0<∏i=1n∫Xbest−Xi−dXbest−Xi+dfxdx<1⟹0<pX+δ∈C1<1.

Because *δ* depends on Gaussian distribution and *p*{(*X* + *δ*) ∈ *C*_1_} is continuous, then

(14) $$\begin{array}{c} p\left\{\;\left(\;X^{\prime }+\delta \;\right)\in S_{1}\;\right\}=\min\left\{\;p\left\{\;\left(\;X+\delta \;\right)\in S_{1}\;\right\}\;\right\}. \end{array}$$

Let *φ* = 1 − *p*{(*X*′ + *δ*) ∈ *S*_1_}; we can obtain following equation by (11) and (14):

(15) pX′+δ∈S1pX+δ∈S1<p2→1=1−p2→2⟹p2→21−pX′+δ∈S1⟹p2→2φ.

**Theorem 3 B.**

If one-step transition probability in ARQEA *p*_2→2_ < *φ*, then ARQEA is global convergence.

**Proof**

For the termination criterion *ε* > 0, when the probability *p*′ = *p*_2→2_ = *p*{|*F*(*X*(*t*)) − *F*(*X*_best_)| ≥ *ε*}, *X*(*t*) cannot satisfy the termination criterion *ε* > 0 after *t* generations; we can obtain (16) by Theorem A:

(16) $$\begin{array}{c} \overset{\infty }{\underset{t=1}{\sum }}p_{t}^{\prime }<\overset{\infty }{\underset{t=1}{\sum }}\varphi \end{array}$$

and 0 < *φ* < 1, so ∑_*t*=1_^*∞*^ *φ* = *φ*/(1 − *φ*); we can get (17) by Lemma A:

(17) $$\begin{array}{c} p\left\{\;\overset{\infty }{\underset{i=1}{⋂}}{⋃}_{t\geq i}\left(\;\left|\;F\left(X\left(t\right)\right)-F\left(X_{best}\right)\;\right|\geq \varepsilon \;\right)\;\right\}=0. \end{array}$$

So lim_*t*→*∞*_ *F*(*X*(*t*)) = *F*(*X*_best_). That is, ARQEA can converge to the optimal solution with probability 1.

## 4. Experiments and Results Analysis

In this section, we investigate the search ability of ARQEA by eight test functions and compare the results with GA, QEA, and DCQGA. And the above optimization algorithms are encoded in MATLAB 2010b and implemented on computer with Intel T3200 CPU and 2 G DDR3 memory using Windows XP.

The test functions are listed in Table 1. The test functions contain trigonometric function, polynomial function, and exponential function in the list. And *f*_1_, *f*_2_, and *f*_3_ are two-dimension multiple hump functions; *f*_4_, *f*_5_, *f*_6_, *f*_7_, and *f*_8_ are high dimension functions. Each test function has only one optimal solution in feasible region, in which the minimum of *f*_1_ and *f*_2_ is 0 at [0,0], the minimum of *f*_3_ is 0 at [1,1], and the minimum of test functions *f*_4_, *f*_5_, *f*_6_, *f*_7_, and *f*_8_ is 0 at [0,0,…, 0]. However, the test functions with high dimension will bring more difficulty in global and local search process.

In this experiment, the population size is 50 for GA, QEA, DCQGA, and ARQEA; the max generation is 500 for two-dimension test functions *f*_1_, *f*_2_, and *f*_3_, and 3000 for high dimension test functions *f*_4_, *f*_5_, *f*_6_, *f*_7_, and *f*_8_. And the coding for GA, QEA, DCQGA, and ARQEA is implemented, respectively, as follows:

(a) GA adopts real coding. The real coding is adapted to every variable in GA. The selection operation employs roulette wheel selection, crossover probability is 0.8, and mutation probability is 0.01. The real coding for chromosome is
  (18) $$\begin{array}{c} \left|\begin{array}{cccccc} x_{1} & x_{2} & \cdots & x_{i} & \cdots & x_{n} \end{array}\right|. \end{array}$$
(b) QEA adopts Q-bit coding. The length of every variable is 12. The updating strategy employs the rotating gate based on check table. The coding for the *i*th individual with *n* variables is
  (19) $$\begin{array}{c} p_{i}=\left[\;\left|\begin{array}{cccc} \alpha_{1}^{1} & \alpha_{1}^{2} & \cdots & \alpha_{1}^{l}\\ \beta_{1}^{1} & \beta_{1}^{2} & \cdots & \beta_{1}^{l} \end{array}\right|\cdots \left|\begin{array}{cccc} \alpha_{n}^{1} & \alpha_{n}^{2} & \cdots & \alpha_{n}^{l}\\ \beta_{n}^{1} & \beta_{n}^{2} & \cdots & \beta_{n}^{l} \end{array}\right|\;\right]. \end{array}$$
(c) DCQGA adopts double chain real-coding scheme. The step size of rotation angle is 0.001*π*; mutation probability is 0.05; the coding for the *i*th individual with *n* variables is
  (20) $$\begin{array}{c} p_{i}=\left[\;\left|\begin{array}{c} \cos\left(t_{i1}\right)\\ \sin\left(t_{i1}\right) \end{array}\right|\left|\begin{array}{c} \cos\left(t_{i2}\right)\\ \sin\left(t_{i2}\right) \end{array}\right|\begin{array}{c} \cdots \\ \cdots \end{array}\left|\begin{array}{c} \cos\left(t_{in}\right)\\ \sin\left(t_{in}\right) \end{array}\right|\;\right], \end{array}$$
 where *p*_*ic*_ = (cos(*t*_*i*1_), cos(*t*_*i*2_),…, cos(*t*_*in*_)) and *p*_*is*_ = (sin(*t*_*i*1_), sin(*t*_*i*2_),…, sin(*t*_*in*_)). *p*_*ic*_ and *p*_*is*_ represent a chromosome pair that maps the unit space of *n* dimensions to the solution space by solution space transformation.
(d) ARQEA adopts real coding based on allele with (1) and (2) and employs hybrid updating strategy to update the alleles and the corresponding probability amplitude.

In this experiment, we run repeatedly the above optimization algorithms for each test function and record the running results (including the optimal values, average values, worst values, and average run times) in different optimization algorithms. The comparison results for different optimization algorithms are shown in Table 2. From the comparison results, we can find that the search accuracy of ARQEA is obviously superior to GA's, QEA's, and DCQGA's in solving continuous optimization problem. In addition, the running time of ARQEA is faster than GA's, QEA's, and DCQGA's as well.

**Figure 3:**
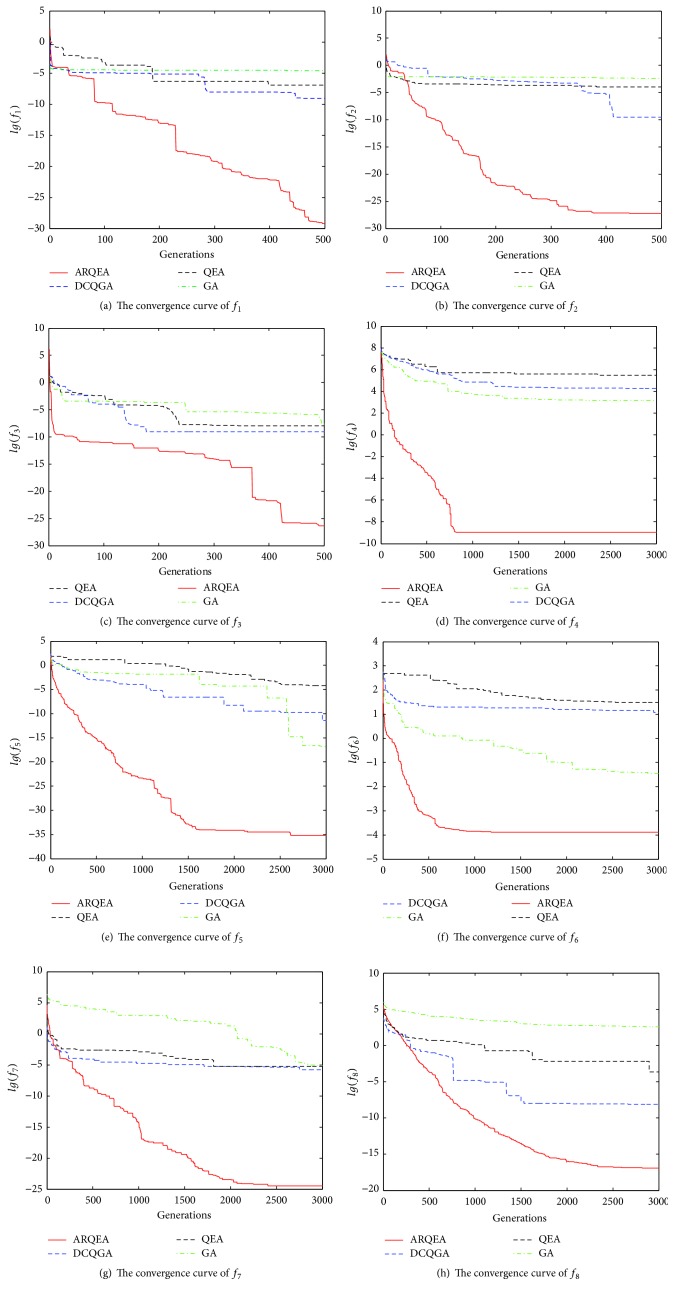
Comparison of convergence for GA, QEA, DCQGA, and ARQEA.

Figure 3 shows the comparison of the convergence optimized by QEA, DCQGA, and ARQEA, respectively. We employ logarithmic ordinate to analyze search accuracy and convergence rate. For two-dimension test functions *f*_1_, *f*_2_, and *f*_3_, the convergence curve of ARQEA shows rapid decline after 500 generations in solving continuous optimization problem. For high dimension test functions, the convergence rate of ARQEA is obviously superior to GA's, QEA's, and DCQGA's, even though the global and local search processes become more difficult. So, due to diversity of allele and hybrid updating strategy, the convergence rate of ARQEA is superior to GA, QEA, and DCQGA.

## 5. Conclusions

In this paper, we present an allele real-coded quantum evolutionary algorithm. The proposed algorithm employs real coding based on probability superposition to maintain the diversity of population and prevent overlong length of chromosome. In hybrid updating strategy, we employ different evolutionary mode for superior and inferior allele in order to balance global search and local search. By comparing with different optimization algorithms, the search accuracy and convergence rate of ARQEA are obviously superior to GA's, QEA's, and DCQGA's in solving continuous optimization problem. For the future, we will analyze the influence on initial population to search accuracy and relationship between parameters in updating strategy and convergence rate.

## Figures and Tables

**Table 1:** The test functions.

Number	Function expression	Search space	*f*_min_
1	f1x1,x2=0.5+sin2⁡x12+x22-0.51+0.001x12+x222∑XX	*x*_*i*_ ∈ [−10,10]	*f*_min_ = 0

2	f2x1,x2=100x1-x222+x1-12∑XX	*x*_*i*_ ∈ [−10,10]	*f*_min_ = 0

3	f3x1,x2=-20exp⁡-0.2x12+x222-expcos⁡2πx1+cos⁡2πx22+exp⁡1∑XX	*x*_*i*_ ∈ [−10,10]	*f*_min_ = 0

4	f4x=418.9829n+∑i=1nxi·sin⁡xi, n=10∑XX	*x*_*i*_ ∈ [−500,500]	*f*_min_ = 0

5	f5x=∑i=1ni+110exp⁡xi-xi-5.5, n=10∑XX	*x*_*i*_ ∈ [−5.12,5.12]	*f*_min_ = 0

6	f6x=14000∑i=1n-1xi2-∏i=1n-1cos⁡xii+1+1, n=20∑XX	*x*_*i*_ ∈ [−100,100]	*f*_min_ = 0

7	f7x=∑i=1nxi2, n=30∑XX	*x*_*i*_ ∈ [−10,10]	*f*_min_ = 0

8	$f_{8}\left(x\right)=10n+\overset{n}{\underset{i=1}{\sum }}x_{i}^{2}-10\overset{n}{\underset{i=1}{\sum }}\cos\left(2\pi x_{i}\right),\;n=30$	*x*_*i*_ ∈ [−10,10]	*f*_min_ = 0

**Table 2:** Comparison with different optimization algorithms.

Test function	Evolutionary algorithms	Best value	Average value	Worst value	Running time (sec.)
*f*_1_	QEA	3.4533*e* − 06	4.5658*e* − 04	1.3433*e* − 03	9.543*e* + 01
	DCQGA	6.8996*e* − 08	4.3543*e* − 06	7.8732*e* − 04	6.526
	GA	2.6704*e* − 11	6.3684*e* − 03	9.7032*e* − 03	8.654
	ARQEA	3.0738*e* − 15	6.0451*e* − 15	2.0368*e* − 14	3.481

*f*_2_	QEA	6.3275*e* − 04	5.2164*e* − 03	1.6233*e* − 02	5.569*e* + 01
	DCQGA	5.5437*e* − 08	1.2654*e* − 06	4.3251*e* − 04	5.658
	GA	1.5684*e* − 05	9.1982*e* − 02	4.0523*e* − 01	6.423
	ARQEA	2.1055*e* − 13	4.5264*e* − 13	7.3382*e* − 13	1.291

*f*_3_	QEA	1.6048*e* − 02	3.2418*e* − 02	5.2529*e* − 02	4.1324*e* + 01
	DCQGA	1.4323*e* − 05	5.2189*e* − 02	1.59171*e* − 01	6.4383
	GA	1.2181*e* − 08	3.7907*e* − 04	6.3203*e* − 03	2.2957
	ARQEA	2.5346*e* − 16	4.8491*e* − 13	2.1526*e* − 09	4.4252

*f*_4_	QEA	5.0412*e* + 01	1.6542*e* + 02	3.47741*e* + 02	4.8342 + 03
	DCQGA	2.4342	7.0424*e* + 01	2.429242*e* + 02	5.3226*e* + 01
	GA	7.0136*e* − 04	2.0751*e* + 01	2.3687*e* + 01	3.5501*e* + 01
	ARQEA	1.2728*e* − 04	1.2728*e* − 04	1.2728*e* − 04	3.1341*e* + 01

*f*_5_	QEA	4.9056*e* − 04	1.3872*e* − 02	3.4341*e* − 02	3.5231*e* + 02
	DCQGA	7.3975*e* − 06	1.8576*e* − 05	3.9103*e* − 03	6.3342*e* + 01
	GA	5.0090*e* − 08	1.4547*e* − 05	6.9581*e* − 05	4.7454*e* + 01
	ARQEA	1.7982*e* − 16	3.2573*e* − 16	8.8818*e* − 16	5.0032*e* + 01

*f*_6_	QEA	2.2667*e* − 01	3.3742	7.4107	3.3703*e* + 03
	DCQGA	3.2541	2.3371	5.7233	1.0399*e* + 02
	GA	2.9913*e* − 03	2.3544*e* − 01	6.627*e* − 01	7.3251*e* + 01
	ARQEA	9.96785*e* − 03	2.0576*e* − 02	2.7094*e* − 02	4.812*e* + 01

*f*_7_	QEA	3.6544*e* − 02	4.7685*e* − 01	1.3276	3.586*e* + 03
	DCQGA	8.9768*e* − 04	3.4765*e* − 03	5.5876*e* − 02	1.635*e* + 02
	GA	6.0000*e* − 03	3.7400*e* − 02	0.2193	6.3324*e* + 01
	ARQEA	4.9223*e* − 14	7.2357*e* − 12	8.5674*e* − 11	5.011*e* + 01

*f*_8_	QEA	6.3267*e* − 03	2.6832*e* − 01	1.9519	1.278*e* + 04
	DCQGA	3.5257*e* − 05	4.2347*e* − 04	7.0863*e* − 01	2.8593*e* + 03
	GA	2.6432*e* − 02	1.4242	3.0274	8.07*e* + 01
	ARQEA	4.1248*e* − 11	5.3442*e* − 10	9.3327*e* − 8	6.158*e* + 01
